# Dual-Targeted Inhibition of PAR_2_ Endosomal Signaling in Synoviocytes and Nociceptors Attenuates Osteoarthritis Pain

**DOI:** 10.3390/nano16171100

**Published:** 2026-09-01

**Authors:** Marcella de A. Ferreira, Paz Duran, Raquel Tonello, Veronica Emad Farag, Mae Stabile D’Ambra, Evan Yuhan Chen, Sher B. Poudel, Kam W. Leong, Dane D. Jensen

**Affiliations:** 1 Pain Research Center, College of Dentistry, New York University, New York, NY 10010, USA; md5988@nyu.edu (M.d.A.F.); rt2368@nyu.edu (R.T.);; 2 Translational Research Center, College of Dentistry, New York University, New York, NY 10010, USA; 3 Department of Molecular Pathobiology, College of Dentistry, New York University, New York, NY 10010, USA; maedambra@gmail.com (M.S.D.); poudelsb03@gmail.com (S.B.P.); 4 Department of Biomedical Engineering, Columbia University, New York, NY 10027, USA; vef2109@columbia.edu (V.E.F.);

**Keywords:** osteoarthritis, pain, PAR_2_, endosomal signaling, nanoparticle

## Abstract

Osteoarthritis (OA) is the most prevalent chronic joint disease characterized by the progressive degeneration of articular cartilage leading to debilitating locomotor restrictions and persistent pain. The pathogenesis of OA is driven by a complex cycle of inflammatory mediators, where G protein-coupled receptor protease-activated receptor 2 (PAR_2_) has emerged as a key driver of both tissue degradation and joint inflammation. PAR_2_ is expressed on various joint tissues, and on the terminals of nociceptive sensory neurons, providing a direct link to structural joint inflammation and chronic nociception. However, the therapeutic potential of PAR_2_ antagonists is hampered by the rapid clearance of drugs from the synovial space. Here, we investigate the therapeutic potential of nanoparticle-mediated drug delivery to achieve sustained PAR_2_ inhibition in OA. OA was induced in C57BL/6 mice via intra-articular injection of sodium iodoacetate, followed by treatment with the PAR_2_ antagonist, AZ3451, encapsulated within PAMAM-Chol nanoparticles (PAMAM-Chol-AZ3451). Our results demonstrate that the PAMAM-Chol-AZ3451 nanoparticles blocked cytokine release from synoviocytes and provided a significantly more sustained and effective antinociceptive effect compared to the free AZ3451, with confirmed nanoparticle uptake by neurons and synoviocytes. Furthermore, cell-specific genetic deletion confirmed that PAR_2_ expressed on Nav1.8 nociceptors is a critical contributor to OA-induced nociception and locomotor deficits. These findings establish that PAR_2_ signaling in neurons is critical for OA nociception and that nanoparticle delivery of PAR_2_ antagonists (PAMAM-Chol-AZ3451) represents an effective strategy for sustained inhibition of PAR_2_ activity, offering a novel approach for managing chronic OA pain.

## 1. Introduction

Osteoarthritis (OA) is the most prevalent chronic joint disease. It is characterized by the progressive degeneration of articular cartilage leading to chronic pain and restricted mobility [1,2,3]. Previously viewed as normal wear and tear from use, OA is now defined as a complex pathology involving abnormal tissue remodeling driven by a robust increase in inflammatory mediators [4,5,6]. The G protein-coupled receptor (GPCR), protease-activated receptor 2 (PAR_2_), sits at the center of this cycle of inflammation, cartilage breakdown and nociception. It acts as a central checkpoint and driver in both structural joint degradation and chronic pain in OA [7,8,9].

PARs are a distinct family of seven-transmembrane domain GPCRs consisting of four members (PAR_1_ to PAR_4_) that are activated by the proteolytic cleavage of their N-terminal domain. PAR_2_ is expressed in joint tissues, including synovium, cartilage, and sensory neurons [9,10], and is upregulated in OA articular cartilage and synovial tissues compared to healthy controls [11]. PAR_2_ is activated by a variety of serine proteases, including matriptase, trypsin, neutrophil elastase and cathepsin S, which are released during inflammation and injury of the joint [12,13]. Following activation, PAR_2_ orchestrates distinct inflammatory signals across different joint tissues [14]. First, within the synovium, PAR_2_ activation on synoviocytes and macrophages generates a cascade of pro-inflammatory cytokines including IL-1β, TNF-α, and IL-6 [7]. These inflammatory signals are associated with an increase in other damage-associated molecular patterns (DAMPs) like cell free DNA (cfDNA) which are linked to increased PAR_2_ expression [15,16]. Second, within cartilage, PAR_2_ activation on cartilage promotes the expression of matrix metalloproteinases (MMPs). MMP expression leads to the depletion of aggrecans and type I collagen [17]. Additionally, PAR_2_ activation on chondrocytes leads to senescence and apoptosis, limiting maintenance and repair of the extracellular matrix [7]. Third, distinct from its role in inflammation and structural changes in the joint, PAR_2_ is expressed on dorsal root ganglia (DRGs) neurons which innervate the knee joint and where PAR_2_ is situated to be the link between inflammation and nociception in OA [10,18].

Although PAR_2_ has been established as a therapeutic target in OA, practical inhibition of PAR_2_ is limited by significant biological barriers. Specifically, poor penetration into the dense cartilage matrix and low retention in the synovial space limit the effectiveness of standard pharmacological treatments. Nanoparticle-mediated delivery is one mechanism that can overcome these limitations. Cationic polyamidoamine (PAMAM) dendrimer-based nanoparticles are a platform that have increased association with cartilage tissue and have demonstrated rapid cellular uptake to the endosomal network in many cell types, including sensory neurons [19,20,21,22]. Furthermore, these cationic polymers possess inherent therapeutic utility through their ability to scavenge anionic inflammatory DAMPs, such as cfDNA, from the synovial space [16].

Herein, we investigated whether encapsulating the potent, specific PAR_2_ antagonist AZ3451 within PAMAM nanoparticles could yield superior therapeutic efficacy compared to the free drug in mitigating OA pathology. Crucially, recent evidence indicates that PAR_2_ continues to signal from endosomal compartments post-internalization, and that this endosomal trafficking is required for a sustained nociceptive response in sensory neurons. We have previously demonstrated that nanoparticles are effective for inhibiting PAR_2_ endosomal signaling [21,22,23,24,25]. While targeting endosomal PAR_2_ using nanoparticle platforms has shown promise in acute pain models, the precise contribution of PAR_2_ endosomal signaling in chronic OA pain remains unclear. By exploiting the endocytic uptake of PAMAM nanoparticles, we demonstrate that PAMAM-encapsulated AZ3451 significantly enhances therapeutic efficacy by simultaneously halting the expression and extracellular release of the cytokine/DAMP cascade in synoviocytes and blocking the intracellular, PAR_2_ endosomal signaling axis in nociceptors. This dual-targeted approach provides a profound, long-lasting analgesic response and a marked reduction in joint inflammation, offering a robust nanotechnology-driven strategy for comprehensive OA management.

## 2. Materials and Methods

### 2.1. Animals

All procedures adhered to the *Guide for the Care and Use of Laboratory Animals* (NIH). The animal study protocol was approved the New York University Institutional Animal Care and Use Committee (PROTO202400027, Approved 3 February 2026). All experiments were performed in male and female C57BL/6 mice (wild type, Cat# 000664, The Jackson Laboratory, Bar Harbor, ME, USA), Par_2_ global knockout (Par_2_^−/−^, Cat# 0004993 The Jackson Laboratory, Bar Harbor, ME, USA) mice, Par_2_Nav1.8 conditional knockout [25] and Par_2_-mugfp [21] mice 8–10 weeks were raised in-house. Following arrival at the animal care facility, mice were allowed to acclimate for at least 1 week, and they were housed in controlled conditions with a 12 h light/dark cycle (lights on at 07:00) and a temperature of 22 ± 2 °C. Mice had ad libitum access to food and water, with weekly cage changes. Mice were housed in groups of 4–5.

Investigators were blinded to the treatment groups. Mice were randomly assigned to treatments groups (www.randomization.com) and the order of treatments was controlled to minimize confounding data. All efforts were made to minimize animal suffering.

### 2.2. PAMAM Synthesis and Drug Administration

For in vivo use, AZ3451, a PAR_2_ antagonist, was encapsulated into polyamidoamine dendrimer (PAMAM) nanoparticles, which were synthesized exactly as described previously [22,25]. PAMAM-Chol-AZ3451 nanoparticles were loaded with 20% AZ3451 (wt/wt%) but had an encapsulation efficiency of >99%. As established previously [22,25], PAMAM-Chol nanoparticles had a hydrodynamic diameter of ~220 nm, a polydispersity index (PDI) of <0.2 and a positive zeta potential of +65 ± 1 mV. Previous studies demonstrated the PAMAM-Chol nanoparticles did not cause cytotoxicity (0.3 to 10 µg/mL) following incubation of to 24 h with HEK293 cells [22]. Passive in vitro AZ3451 release kinetics were previously demonstrated as AZ3451 was steadily released for at least 48 h. PAMAM-Chol-AZ (10 µM), free AZ3451 (10 µM), empty nanoparticles (PAMAM-Chol-Ø) or vehicle (PBS) was administered intra-articular at the knee joint route in a volume of 10 µL per mouse.

### 2.3. Induction of OA Model

On the day of injection, a fresh solution of monosodium iodoacetate (MIA) Bioultra (1 mg/kg; Sigma 57858-25 g) in sterile saline was prepared. Mice were anesthetized in an induction chamber using isoflurane 2.5% mixed with O_2_ at a flow rate of 1.5 L/min and maintained with face mask at 2.0% during the injection, and the anesthesia was confirmed by the absence of a withdrawal response to a hind paw pinch. The mice were put on the back in dorsal recumbency, the skin around the knee was shaved and disinfected with a 70% alcohol pad. A 26-G needle attached to a Hamilton syringe was positioned horizontally along the knee to locate the gap beneath the patella. Gentle pressure was applied to mark the injection site, after which the needle and syringe were lifted vertically. The needle was then inserted at the marked site, passing through the patellar tendon perpendicular to the tibia for the intra-articular injection (10 µL) [26]. Control mice received an equivalent injection of sterile saline.

### 2.4. Study Design

OA model was induced on day 0 and for the model behavior were assessed before and on days 1, 3, 7, 10, 14, 21 and 28 after OA induction. To investigate PAR_2_-mediated signaling, animals received an intra-articular injection into the knee joint 7 days after OA induction with either PAMAM-Chol nanoparticles containing the PAR_2_ antagonist AZ3451 (PAMAM-Chol-AZ3451, 10 µM), empty nanoparticles (PAMAM-Chol-Ø), or free AZ3451 (10 µM). For the treatment, paw mechanical thresholds (PWT) and weight-bearing was assessed up to 28 days after OA induction. Spontaneous nociception and potential adverse effects were also evaluated using a behavioral spectrometer at day 8 after OA induction. For PAR_2_ knockout mice, the OA model was induced and the behavioral measurements were assessed before and on days 1, 3, 7, 10, 14, 21 and 28 after OA induction.

### 2.5. Behavioral Testing

#### 2.5.1. Mechanical Allodynia

Mechanical allodynia was assessed by measuring hind paw withdrawal thresholds in response to von Frey filament stimulation using the up-and-down method [27]. Mice were acclimatized to the testing apparatus, consisting of individual clear Plexiglas boxes on an elevated wire mesh platform, for 1 h daily over 2 days to allow access to the plantar surface of the hind paws. A series of von Frey filaments (0.008, 0.02, 0.07, 0.16, 0.4, 1.0, and 2 g; Stoelting, Wood Dale, IL, USA) were applied perpendicularly to the plantar surface of each hind paw. A positive response was defined as a clear paw withdrawal or shaking. The next weaker filament was applied following a positive response, and the next stronger filament was applied following a negative response. This process consisted of six measurements and the paw withdrawal thresholds were analyzed as the log transforms of the von Frey force [28].

#### 2.5.2. Functional Impairment—Static Weight-Bearing, WB

Static weight-bearing distribution was evaluated to assess the functional impact of the injury [26,29]. Hindlimb weight-bearing was quantified using a Bioseb Incapacitance Tester (Bioseb, Vitrolles, France), which measures the weight supported by each hindlimb of a stationary animal. Prior to model induction, mice were habituated and trained to the testing paradigm during short sessions over two days to ensure comfort. For each measurement, three readings were collected and averaged per animal. The weight borne by the ipsilateral (injured) limb was expressed as a percentage of the total weight borne by both hindlimbs (WB% = [ipsilateral limb weight total hindlimb weight] × 100%). Measurements were recorded at baseline (before OA induction), and on days 1 to 28 post OA induction.

#### 2.5.3. Non-Evoked Behavior

Spontaneous, non-evoked behavior of mice was assessed using a behavioral spectrometer (Behavioral Instruments, Hillsborough, NJ, USA), a validated and unbiased instrument for rodent phenotyping [30,31]. This system has been previously employed to study aberrant behaviors in mouse models of autism, restraint stress, and various pain states (inflammatory, neuropathic, and visceral pain). The spectrometer consisted of a 40 cm^2^ arena equipped with a ceiling-mounted CCD camera and a front-located door aperture. Locomotion was monitored by a floor vibration sensor and 32 wall-mounted infrared beam arrays. Individual mice were placed in the center of the arena, and their behavior was recorded for 20 min. Analysis involved a combination of video tracking (Viewer3, BiObserve, Sankt Augustin, Germany) and vibration analysis. The following parameters were quantified as previously described: total distance traveled, number of central area visits, average velocity, track length, ambulation (% activity), time spent grooming, and wall distance.

#### 2.5.4. Collection of Mouse Tissue

Mice were anesthetized with 5% isoflurane and transcardially perfused with PBS followed by 4% paraformaldehyde (PFA) in PBS. Left knee joints were collected, postfixed in 10% formalin in PBS at 4 °C overnight. The knees were decalcified in a 10% EDTA solution at 4 °C for 4 weeks and embedded in OCT compound. Frozen sections (10 µm) were cut, mounted onto Superfrost Plus slides (Fisher Scientific, Pittsburg, PA, USA), air-dried for 15 min, and stored at −20 °C.

#### 2.5.5. PAMAM-Chol-Cy5 Nanoparticle Uptake in the Joint Knee

Par_2_-muGFP mice were lightly anesthetized (isoflurane) and shaved along the knee. PAMAM-Chol loaded with the fluorescent dyes, cyanine 5 (Cy5), were injected intra-articularly (10 µL). Mice were returned to their home cage and allowed to recover for 30 min. Mice were then anesthetized and sacrificed (isoflurane), perfused with ice cold PBS, and 4% paraformaldehyde and the knees were processed as stated earlier. The knees were sectioned (10 μm), labeled via in situ hybridization and were incubated overnight at 4 °C with primary antibodies targeting GFP (1:200; cat#600-401-215L, Rockland Immunochemicals, Limerick, PA, USA) or β-III tubulin (1:200; cat#D71G9, Cell Signaling Technology, Danvers, MA, USA). Slides were washed and incubated with goat anti-rabbit Alexa Fluor 488 (1:1000; cat#A32790, Invitrogen, Waltham, MA, USA) (1 h, RT). Slides were washed and incubated with DAPI, 49,6-diamidino-2-phenylindole (1 mg/mL, 5 min) and mounted with ProLong^®^ Glass Antifade (cat#P36980, Thermo Fisher, Pittsburg, PA, USA). Sections were imaged on the Leica DMi8 Widefield microscope with HCX PL APO 10× objective and 40× objective to determine the uptake and retention of PAMAM-Chol-Cy5 in the knee sections.

Co-localization between PAMAM-Chol-Cy5 nanoparticles with either PAR_2_-GFP or β-III tubulin labeled-neurons was quantified using the JaCOP plugin in ImageJ (v1.54p) [32]. Channel thresholds were determined using the Otsu method and the Manders’ coefficients were calculated.

#### 2.5.6. Cell Lines

Human SW982 cells (female-derived) obtained from ATCC (HTB-93^TM^, Manassas, VA, USA) were maintained in culture with Leibovitz’s L-15 Medium supplemented with 10% fetal bovine serum (FBS) and penicillin/streptomycin, 50 IU/mL at 37 °C with no CO_2_.

#### 2.5.7. PAMAM-Chol Nanoparticle Uptake

SW982 cells were plated on poly-D-lysine coated 12 mm cover slips in a 24-well plate. 24 h after plating, cells were treated with PAMAM-Chol-Cy5-loaded nanoparticles for 2 or 4 h at 37 °C. Cells were washed with PBS and fixed with PFA (4% paraformaldehyde, in PBS, 20 min, in ice). Cells were blocked in 5% normal horse serum (NHS), 0.1% Saponin in PBS (1 h, RT), and incubated with rabbit anti-EEA1 (1:1000; cat#PA1-063A, Invitrogen) overnight, 4 °C. Cells were washed and incubated with anti-rabbit Alexa Fluor^®^ 488 (1:1000, 1 h, RT; cat# A-11008, Invitrogen). Cover slips were washed 3× with PBS and mounted on glass slides with ProLong^®^ Glass Antifade Mountant (cat#P36980; Thermo Fisher). Cells were imaged using a Leica SP8 confocal microscope with HCX PL APO 63× (NA 1.40) oil objectives (Leica-Microsystems, Wetzlar, Germany). Images were processed using ImageJ and figures were made in Adobe Illustrator (v 30.7).

#### 2.5.8. Calcium Imaging

SW982 cells were plated in a 96-well plate. Twenty-four hours after plating, cells were loaded with Fura2-AM ester (1μM) in calcium buffer [150 mM NaCl, 2.6 mM KCl, 1.18 mM MgCl_2_, 2.2 mM CaCl_2_, 10 mM Glucose, 10 mM HEPES, and 0.5% BSA] supplemented with probenecid (0.04 mM) and pluronic acid (0.5 μM) for 60 min at 37 °C. Changes in fluorescence, which are proportional to [Ca^2+^]i were measured at 340/380 nm excitation and 530 nm emission wavelengths using a FlexStation III plate reader (Molecular Devices, San Jose, CA, USA). Baseline measurements were recorded prior to agonist addition. Cells were incubated for 30 min with inhibitors prior to agonist administration.

#### 2.5.9. Cytokine Assay

SW982 cells were plated in a 24-well plate and incubated for 48 h. Media was replaced and the cells were serum starved for 16 h then incubated with either vehicle, free AZ3451 (10 µM) or PAMAM-Chol-AZ3451 (10 µM) for 2 h. Cells were washed and media was replaced. Cells were challenged with either PBS (vehicle control) or the PAR_2_ agonist, 2-Furoyl-LIGRLO (10 µM) for 24 h. The conditioned media was removed, flash frozen, and stored at −80° C. PAR2-induced cytokine release was quantified using the Proteome Profiler Human Cytokine Array (cat#ARY005B, R&D Systems, Minneapolis, MN, USA) following manufacturer’s protocols, substituting Streptavidin Alexa-790 (cat#S11378, Invitrogen) for the kit streptavidin-HRP. Membranes were imaged using the Amersham Typhoon with the IR Long (785 nm) laser. Cytokine intensity was quantified with ImageJ (NIH), membrane values were normalized across experiments via the reference spots and to the PBS control supernatant.

#### 2.5.10. Statistics

Data are presented as mean ± SEM. Groups of 6 mice per condition (*n* = 6) were analyzed. For two-group comparisons, statistical significance was determined using a two-tailed Student’s *t*-test. For multiple group comparisons, one-way or two-way analysis of variance (ANOVA) was performed, followed by appropriate post hoc tests (Sidak, Tukey, Newman–Keuls, or Dunnett) to identify specific group differences. To meet the parametric assumptions, mechanical hyperalgesia was log transformed prior to statistical analysis (log_10_). *p* < 0.05 was considered statistically significant. Specific sample sizes and statistical tests used for each experiment are detailed in the respective figure legends.

## 3. Results

### 3.1. Nanoparticle-Mediated Delivery of PAR_2_ Inhibitor Attenuates OA Nociception

To investigate the contribution of endosomal PAR_2_ signaling to OA pain, we utilized a pharmacological approach. Previous studies showed that nanoparticle-encapsulated drugs are more effective than free drugs in blocking PAR_2_-mediated signaling [23] and in targeting pain associated with head/neck cancer [33,34]. This led us to encapsulate the specific PAR_2_ negative allosteric modulator AZ3451 into PAMAM dendrimer nanoparticles (PAMAM-Chol-AZ3451). This formulation was designed to target both membrane-bound and endosomally trafficked PAR_2_, preventing PAR_2_ from activating and initiating the signaling pathways, such as Gα and β-arrestin recruitment.

**Figure 1:**
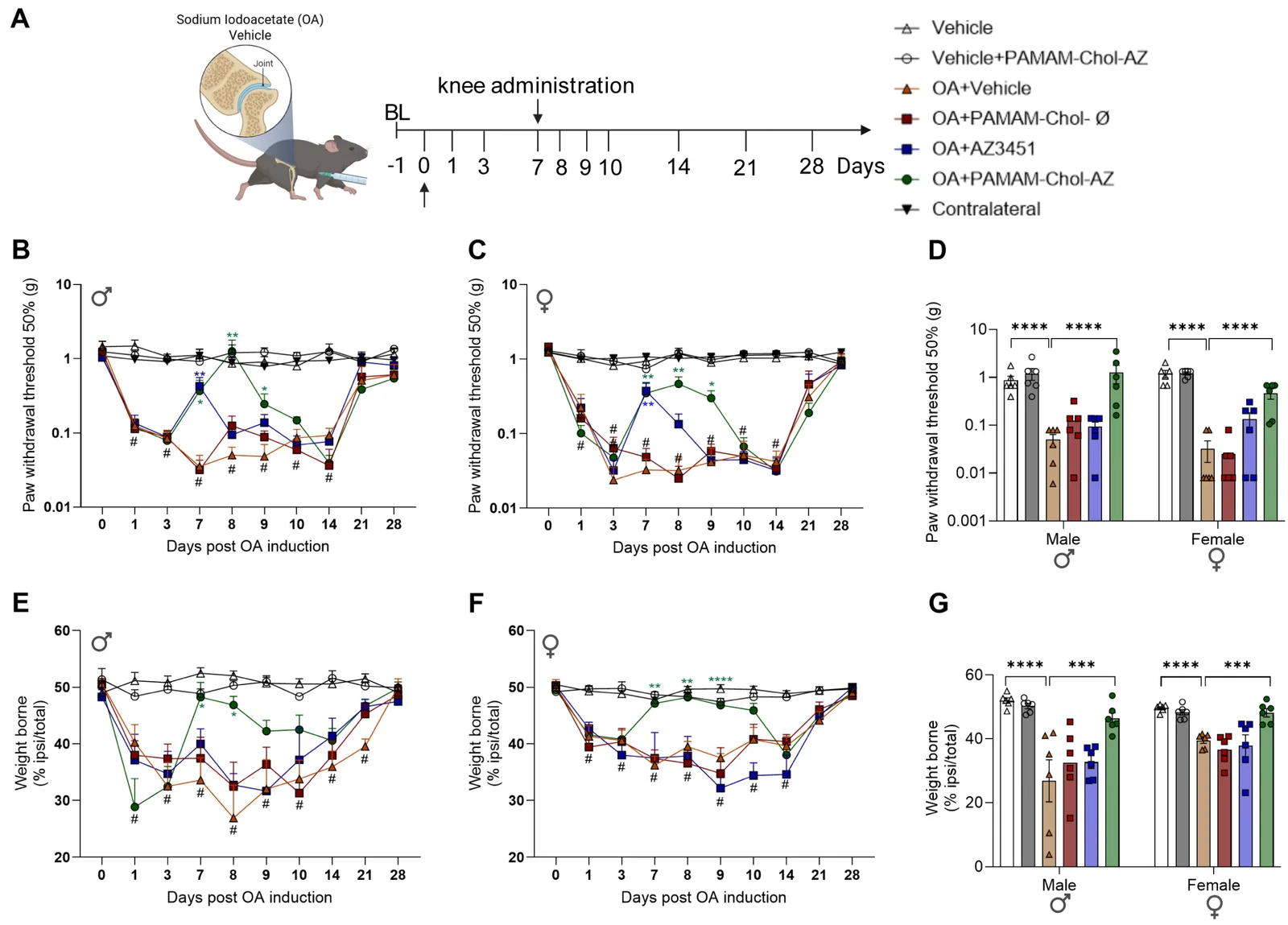
Nanoparticles antagonizing PAR_2_ reversed nociception induced by OA model in mice. (**A**) Experimental timeline. (**B**,**C**) Paw-withdrawal threshold was measured before (day 0, arrow) and after OA induction in both male (**B**) and female (**C**) mice. The analgesic effects of an intra-articular injections of PAMAM-Chol-nanoparticles containing the PAR_2_ antagonist AZ3451 (PAMAM-Chol-AZ, 10 µM), empty nanoparticles (PAMAM-Chol-Ø), free AZ3451 (10 µM), or vehicle were measured daily after administration at day 7 post-OA induction. (**D**) Paw-withdrawal threshold at day 8 (24 h after treatment) for male and female mice. (**E**,**F**) Weight-bearing deficit was measured before (day 0) and after OA induction in both male (**E**) and female (**F**) mice. The analgesic effects of an intra-articular injections of PAMAM-Chol-nanoparticles containing the PAR_2_ antagonist AZ3451 (PAMAM-Chol-AZ, 10 µM), empty nanoparticles (PAMAM-Chol-Ø), free AZ3451 (10 µM), or vehicle were measured daily after administration at day 7 post-OA induction. (**G**) Weight-bearing deficit at day 8 (24 h after treatment) for male and female mice. *n* = 6 mice/group. Data are expressed as the mean ± SEM. For time-line Figures (**B**,**C**,**E**,**F**), data analyzed by 2-way ANOVA with Dunnett’s multiple comparison test to determine difference between groups and Tukey’s test was used to check difference with baseline. For Figures (**D**,**G**), data analyzed by 2-way ANOVA with Tukey’s multiple-comparison test to determine differences between groups. * *p* ≤ 0.05, ** *p* ≤ 0.01, *** *p* ≤ 0.001, **** *p* ≤ 0.0001 when compared with OA + Vehicle (orange lines and columns) and # *p* ≤ 0.05 when compared with baseline.

Following the induction of OA via intra-articular injection of MIA into the left knee (Figure 1A), mice developed mechanical allodynia starting on day 1, peaking between day 7 and 14, and returning to baseline by day 21–28 (Figure 1B,C). On day 7, at peak nociception, mice received a single intra-articular injection of vehicle, AZ3451 (10 µM/10 µL), PAMAM-Chol-AZ3451 (10 µM/10 µL), or empty nanoparticles (PAMAM-Chol-Ø, Figure 1A). PAMAM-Chol-AZ3451-loaded nanoparticles achieved a profound and prolonged reduction in OA-induced mechanical allodynia in both male (Figure 1B) and in female (Figure 1C) mice from 2 to 48 h after treatment (from day 7 to 9 after OA-induction), while the free AZ3451 effect lasted just hours after injection (day 7 after OA-induction). The peak antinociceptive effect of PAMAM-Chol-AZ3451-loaded nanoparticle was observed 24 h after treatment (day 8 after OA-induction) with no significant differences in efficacy between sexes at this time point (Figure 1D).

To check functional impact of the injury, we used the weight-bearing-deficit test, where mice avoid putting weight in the injured ipsilateral limb side and naïve mice trend to have a 50% balance. Similar to mechanical allodynia, the OA model induced a significant weight-bearing deficit, as evidenced by the decrease in weight borne on the injured leg (<50%) since day 1 in both male (Figure 1E) and female mice (Figure 1F). Remarkably, PAMAM-Chol-AZ3451 significantly attenuated the weight-bearing asymmetry from 2 to 24 h in male mice (Figure 1E) and from 2 to 48 h in female mice, while free AZ3451 completely failed to reverse this functional deficit (Figure 1F). At the 24 h peak effect, both sexes exhibited a comparable degree of therapeutic reversal (Figure 1G), though the formulation demonstrated a significantly longer effect in female mice.

**Figure 2:**
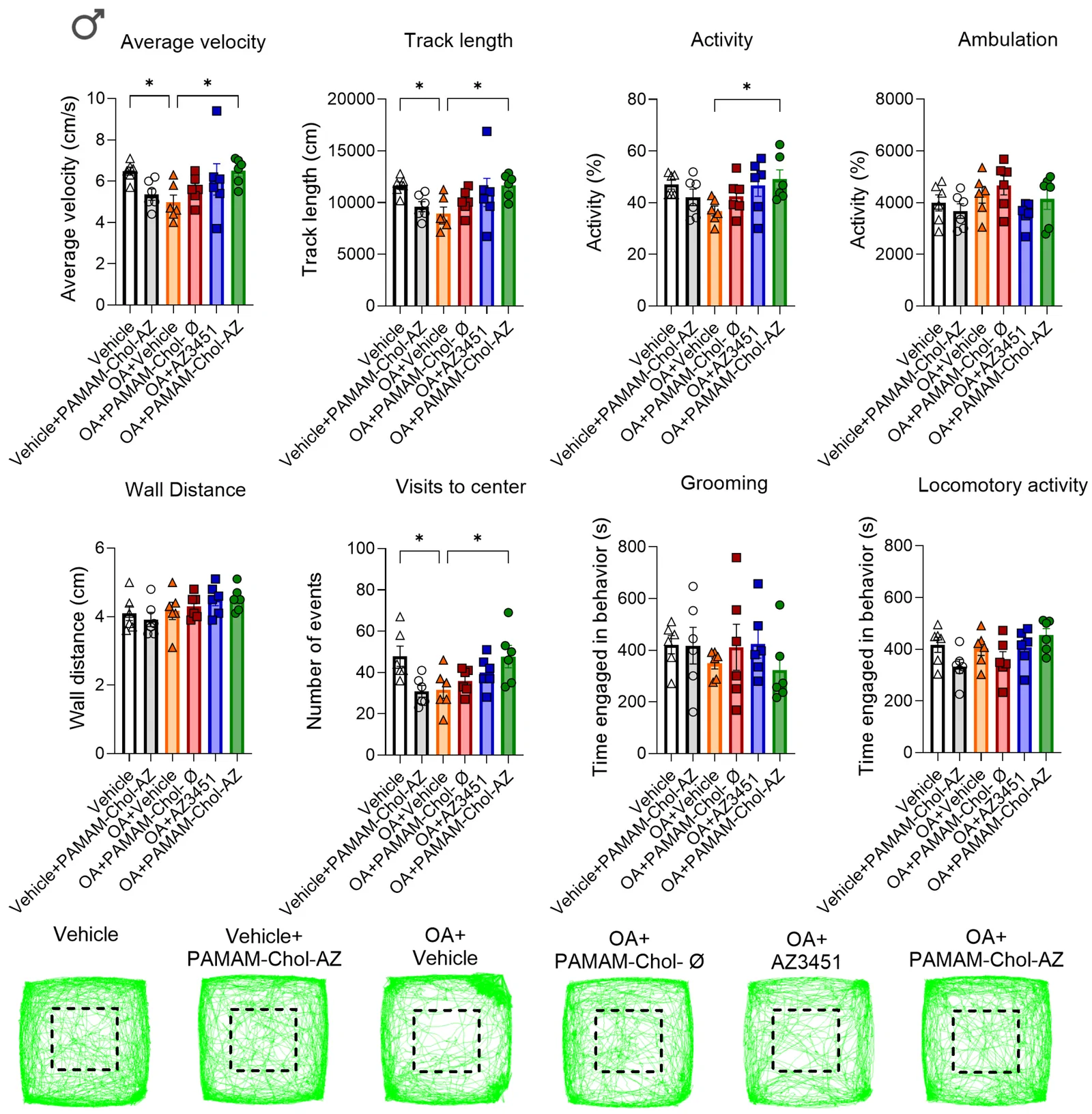
Nanoparticles antagonizing PAR_2_ effect in non-evoked behaviors induced by OA in male mice. Non-evoked nociceptive behavior in OA male mice recorded for 30 min 24 h after administration of PAMAM-Chol-nanoparticles containing the PAR_2_ antagonist AZ3451 (PAMAM-Chol-AZ, 10 µM), empty nanoparticles (PAMAM-Chol-Ø), free AZ3451 (10 µM), or vehicle (8 days after OA induction). Visits to center area marked by black square and representative images of the track records are shown. *n* = 6 mice/group. Data are expressed as the mean ± SEM. Data analyzed by 1-way ANOVA with Dunnett’s multiple-comparison test to determine differences between groups. * *p* ≤ 0.05, when compared with OA + Vehicle (orange columns).

**Figure 3:**
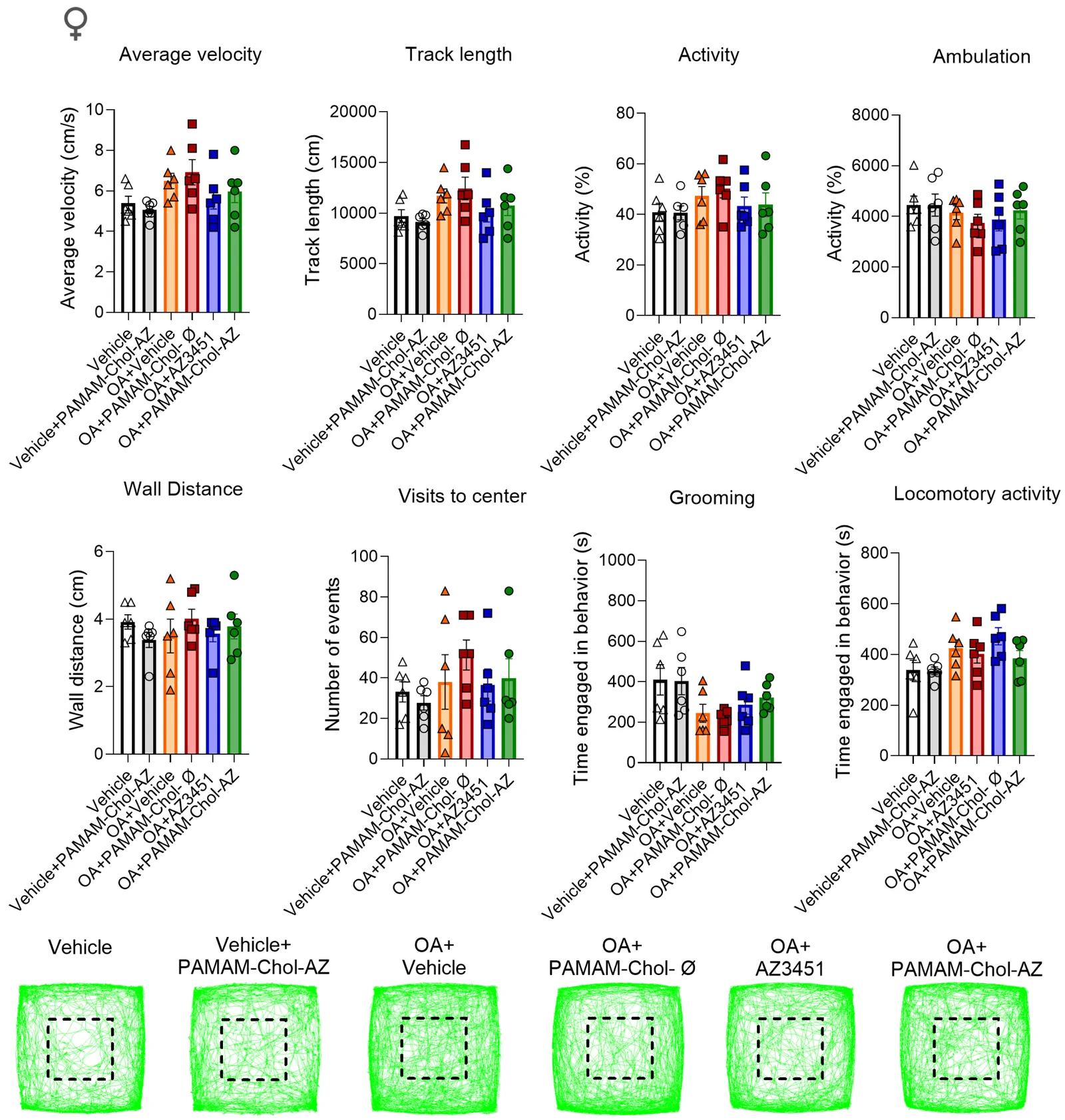
Nanoparticles antagonizing PAR_2_ effect in non-evoked behaviors induced by OA in female mice. Non-evoked nociceptive behavior in OA female mice recorded for 30 min 24 h after administration of PAMAM-Chol-nanoparticles containing the PAR_2_ antagonist AZ3451 (PAMAM-Chol-AZ, 10 µM), empty nanoparticles (PAMAM-Chol-Ø), free AZ3451 (10 µM), or vehicle (8 days after OA induction). Visits to center area marked by black square and representative images of the track records are shown. *n* = 6 mice/group. Data are expressed as the mean ± SEM. Data analyzed by 1-way ANOVA with Dunnett’s multiple-comparison test to determine differences between groups.

To assess the impact of OA and treatment on spontaneous locomotor activity and anxiety-like behaviors, mice were evaluated in a behavioral spectrometer 24 h post-treatment (day 8 after OA-induction). In male mice, the OA model significantly reduced parameters such as average velocity, track length, and visits to the center, all of which were reversed by PAMAM-Chol-AZ3451-loaded nanoparticles. Furthermore, PAMAM-Chol-AZ3451 treatment mitigated the OA-induced reduction in activity (Figure 2). The same experimental protocol was used in female mice. However, in female mice, the OA model did not elicit any statistically significant changes in any locomotor activity nor in anxiety-like behaviors across the parameters analyzed (Figure 3). In conjunction with previous studies that demonstrated endosomal signaling of PAR_2_ in models of oral cancer pain and colonic inflammation could be targeted with PAMAM-Chol nanoparticles [21,22,25], this data suggest that endocytosis of PAMAM nanoparticles by cells and neurons in the joint enhances PAR_2_ inhibition and blocks OA-induced nociception.

### 3.2. PAR_2_ Endosomal Signaling in Synoviocytes Contributes to OA Inflammation via Cytokine Release

**Figure 4:**
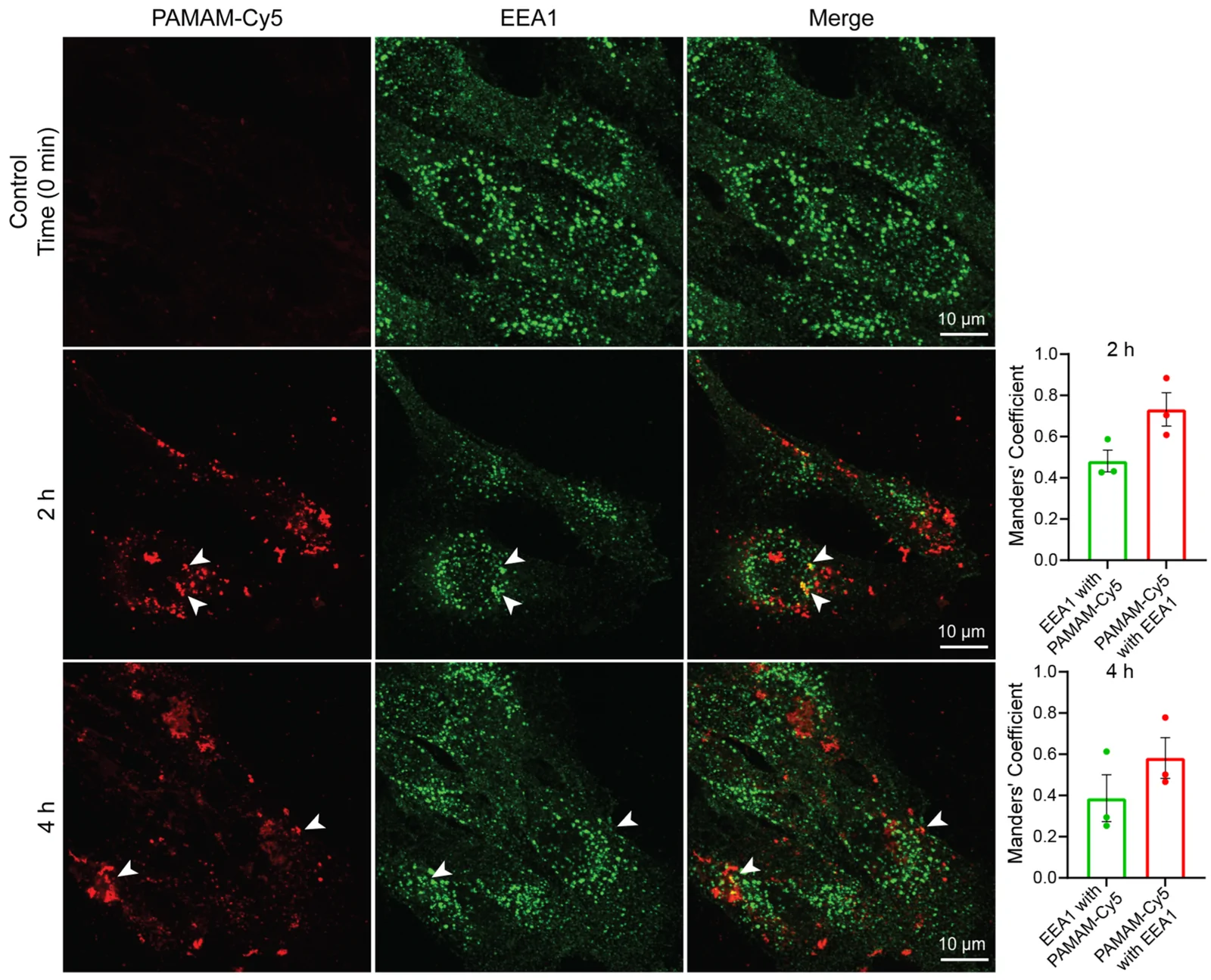
PAMAM nanoparticles are endocytosed by SW982 synoviocytes. PAMAM-Cy5-loaded nanoparticles (red channel) were co-localized with EEA1 (green channel) following 2 and 4 h incubation (arrowheads). Mander’s coefficient shows ~70% of the PAMAM-Chol-Cy5 nanoparticles were co-localized with the early endosomal marker EEA1 following 2 h of incubation. Scale 10 µm, representative images, *n* = 3 independent experiments.

After showing the importance of PAR_2_ endosomal signaling to OA development, we asked which PAR_2_-expressing cells contribute to OA-induced nociception. Given that synoviocytes are primary drivers of OA progression, we first evaluated the capacity of SW982 synoviocytes to internalize PAMAM nanoparticles to target endosomal compartments. Cells were incubated with PAMAM-Chol nanoparticles loaded with the fluorescent dye, cyanine 5 (PAMAM-Chol-Cy5), for 2 and 4 h. The early endosome vesicles were co-stained using an antibody against early endosome antigen 1 (EEA1). Nanoparticle uptake and their co-localization with early endosomes was detected by immunostaining and fluorescence microscopy. Confocal microscopy images revealed that the PAMAM-Chol-Cy5-loaded nanoparticles were efficiently internalized by the SW982 cells, similar to previous studies [25]. Furthermore, co-localization with EEA-1-positive early endosomes was observed as early as 2 h post-incubation, (Figure 4, arrow heads). This co-localization was still evident, though to a lesser extent, at the 4 h time point, suggesting that the nanoparticles were beginning to traffic out of the early endosomes.

**Figure 5:**
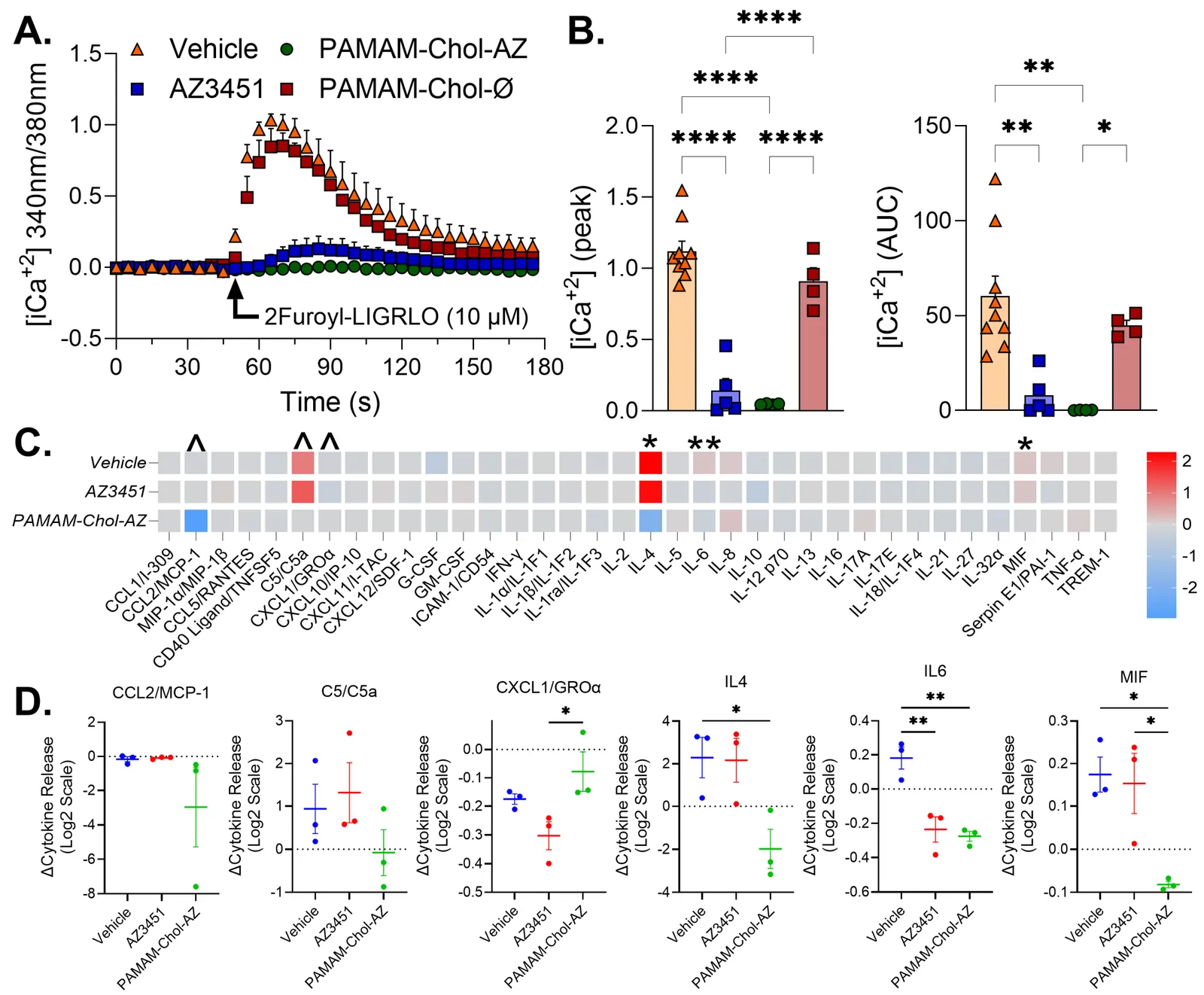
SW982 synoviocytes express functional PAR_2_. (**A**) Kinetics of intracellular calcium mobilization ([iCa^2+^] ratio 340 nm/380 nm) in SW982 cells after stimulation with PAR_2_ agonist 2Furoyl-LIGRLO-NH2 (10 µM, arrow). (**B**) Bar graphs of average peak [iCa^2+^] and total integrated calcium response (AUC) in cells pre-incubated 30 min with vehicle, the PAR_2_ antagonist AZ3451 (10 µM), PAMAM-AZ3451-loaded nanoparticles (10 µM), or empty PAMAM nanoparticles (**B**). Data are expressed as the mean ± SEM. Data analyzed by 1-way ANOVA with Tukey’s multiple-comparison. n ≥ 5, * *p* ≤ 0.05, ** *p* ≤ 0.01, **** *p* ≤ 0.0001. (**C**) Heatmap of differential cytokine and chemokine expression profiles in SW982 cells pretreated with vehicle, AZ3451 (10 µM or PAMAM-Chol-AZ3451 (10 µM) for 2 h and challenged for 24 h with 2Furoyl-LIGRLO (10µM). Color intensity represents the Log2 fold change in protein release compared to non-stimulated (PBS) cells. (**D**) Quantification of cytokine release compared to vehicle control. Data are expressed as the mean ± SEM. Data analyzed by 1-way ANOVA with Tukey’s multiple-comparison test to determine differences between groups. *n* = 3, ^ *p* ≤ 0.1, * *p* ≤ 0.05, ** *p* ≤ 0.01.

Given that PAR_2_ activation is known to recruit and activate Gαq, we decided to perform calcium imaging analysis to confirm the endogenous expression of PAR_2_ in synoviocytes. For this, SW982 cells were loaded with the calcium indicator Fura-2 AM, and changes in fluorescence, indicative of [iCa^2+^] were measured. Cells were pre-incubated with the specific PAR_2_ antagonist AZ3451 (10 µM) for 30 min before stimulation with the PAR_2_ agonist 2Furoyl-LIGRLO-NH2 (2F; 10 µM). Results showed that 2F rapidly increased [iCa^2+^], which was significantly blocked by pre-incubation with AZ3451 (Figure 5A,B).

Once PAR_2_ expression and function were confirmed, we determined whether PAMAM nanoparticle delivery of AZ3451 to endosomal compartments could inhibit PAR_2_ signaling in SW982 cells. SW982 cells were incubated with PAMAM-Chol-AZ3451-loaded nanoparticles (10 µM) or empty PAMAM nanoparticles for 30 min and were then challenged with 2F (10 µM). In empty-PAMAM-treated cells, 2F caused an increase in [iCa^2+^], similar to the untreated cells. However, pre-incubation with PAMAM-Chol-AZ3451-loaded nanoparticles completely prevented 2F-stimulated changes in [iCa^2+^] (Figure 5A,B). Notably, this inhibitory effect was comparable to that observed when using the free AZ3451.

Additionally, SW982 cells have been used to study the release of cytokines and chemokines, inflammatory markers that are key components of arthritis [35]. We used a cytokine array to characterize cytokine and chemokine release from SW982 following PAR_2_ activation. Incubation of SW982 cells with 2F for 24 h resulted in the increased release of inflammatory cytokines and chemokines (Figure 5C). The 2 h pretreatment followed by washout of AZ3451 resulted in a significant reduction in IL-6 release (Figure 5C,D). PAMA-Chol-AZ3451 pretreatment significantly altered pro-inflammatory cytokine and chemokine release, with a significant reduction in IL-4, IL-6 and macrophage migration inhibitory factor (MIF) release and a substantial but not significant reduction in monocyte chemoattractant protein-1 (CCL2/MCP-1, *p* = 0.08) and C5/C5a (*p* = 0.1) release (Figure 5C,D). Additionally, PAMAM-Chol-AZ3451 resulted in an increase in neutrophil chemoattractant CXCL1/GROa compared to free AZ3451 but not the vehicle treated cells (Figure 5C,D). Overall, these results demonstrate that SW982 synoviocytes express functional PAR_2_ and that PAR_2_ activation can drive inflammatory cytokine release. These data also demonstrate that PAMAM-Chol-AZ3451-loaded nanoparticles are more effective than free AZ3451 at inhibiting PAR_2_ signaling and blocking cytokine release which drives joint inflammation in OA.

### 3.3. PAR_2_ Endosomal Signaling in Sensory Neurons Mediates OA Nociception

**Figure 6:**
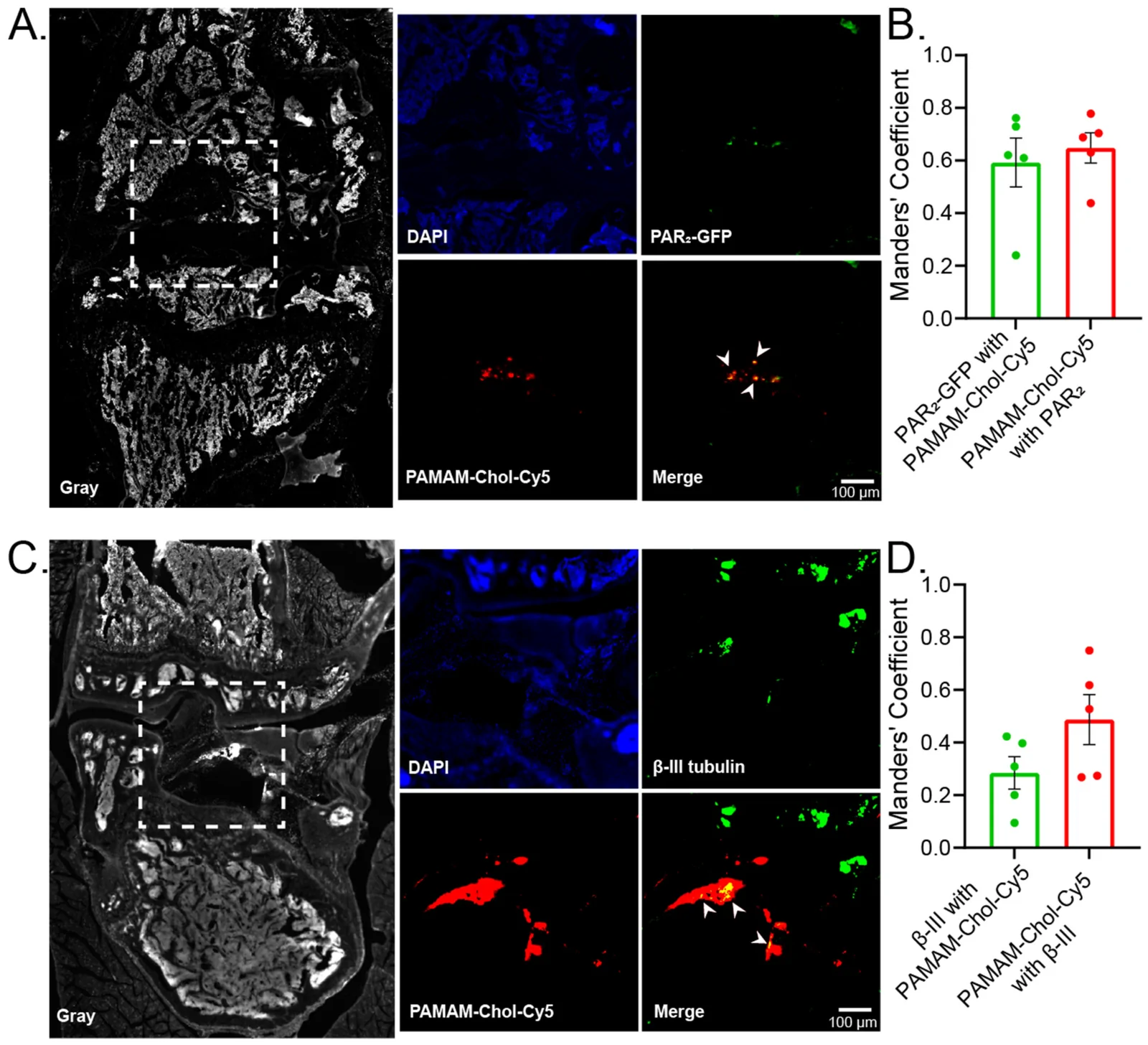
Uptake of PAMAM-Chol-Cy5 nanoparticles within the knee joint. Representative histological images from coronal sections of the knee joint of Par_2_-muGFP mice injected with PAMAM-Cy5 nanoparticles. Localization of PAMAM-Cy5 nanoparticle injection within PAR_2_-GFP (**A**) and within neuronal marker (β-III tubulin, (**C**)) into knee joint. Dashed box marks expanded region. White arrows indicate co-localization of PAMAM-Cy5 nanoparticles within PAR_2_-GFP cells or within neuronal cells. Manders’ overlap coefficients for PAR2-GFP with PAMAM-Chol-Cy5 (**B**) and the neuronal marker β-III with PAMAM-Chol-Cy5 (**D**). Scale bar 100 µm for gray marker. Representative image of *n* = 5 experiments.

To address the neuronal PAR_2_ contribution to OA pathogenesis, we administered intra-articular injection of PAMAM-Chol-Cy5 nanoparticles into PAR_2_-muGFP reporter mice. Subsequent immunohistochemical analysis was performed to verify nanoparticle uptake within the PAR_2_ positive cells and neuronal compartments of the knee. We could verify the intra-articular internalization of PAMAM-Chol-Cy5 into the knee-joint cavity in cells that express PAR_2_, positive for GFP (Figure 6A). The quantification of co-localization showed ~59% of PAR_2_-GFP neurons contained PAMAM-CholCy5 and ~64% of the PAMAM-Chol-Cy5 were present in PAR_2_-GFP positive neurons (Figure 6B). Furthermore, using a neuronal marker (β-III tubulin), we could confirm the uptake of PAMAM-Chol-Cy5 nanoparticles by neurons that innervate the knee cavity (Figure 6C) with ~28% of β-III positive neurons containing nanoparticles and ~48% of the PAMAM-Chol-Cy5 nanoparticles in β-III positive neurons (Figure 6D). These results demonstrate that PAMAM-Chol-Cy5 nanoparticles are taken up by both PAR_2_ positive cells and neurons within the knee. Collectively, these findings suggest that PAR_2_ endosomal signaling plays a crucial role in activating the synoviocytes that establish the inflammatory state of the knee following insult and in the sensory neurons that relay OA nociception.

**Figure 7:**
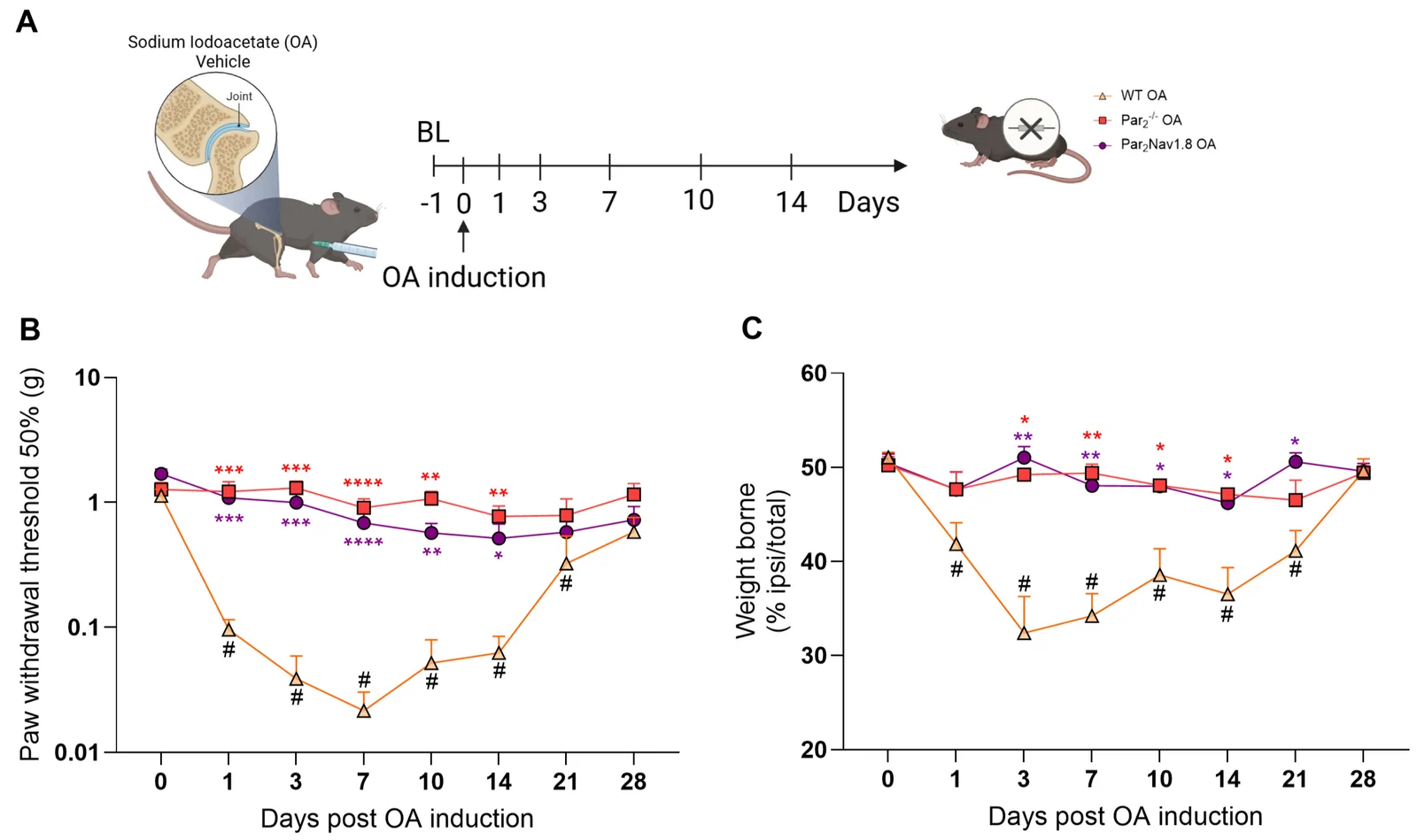
PAR_2_ participation in nociception induced by OA model in mice. (**A**) Experimental timeline. (**B**) Paw-withdrawal threshold was measured before (day 0) and after OA induction in wild type (WT), PAR_2_ global knockout (Par_2_^−/−^) and in mice lacking expression of PAR_2_ in Nav1.8 expressing neurons (Par_2_Nav1.8). (**C**) Weight-bearing deficit was measured before (day 0) and after OA induction in wild type (WT), PAR_2_ global knockout (Par_2_^−/−^) and in mice lacking expression of PAR_2_ in Nav1.8 expressing neurons (Par_2_Nav1.8). *n* = 6 mice/group. Data are expressed as the mean ± SEM. Data analyzed by 2-way ANOVA with Dunnett’s multiple comparison test to determine difference between groups and Tukey’s test was used to check difference with baseline. * *p* ≤ 0.05, ** *p* ≤ 0.01, *** *p* ≤ 0.001, **** *p* ≤ 0.0001 when compared with WT-OA group and # *p* ≤ 0.05 when compared with baseline.

To confirm the contribution of neuronal PAR_2_ to OA pain, the OA model was induced as described before, in wild type (WT), PAR_2_ global knockout (Par2^−/−^) and in mice lacking expression of PAR_2_ in Nav1.8 sensory neurons (Par_2_Nav1.8) (Figure 7A). As before, OA induction increased mechanical allodynia on day 1 in WT mice, evidenced by the decrease in paw withdrawal threshold. OA-induced mechanical allodynia was significantly attenuated in both Par_2_^−/−^ global knockout and Par_2_Nav1.8 conditional knockout mice, until the mice recovered around day 14 post OA induction (Figure 7B).

Additionally, the OA model induced weight-bearing deficit, as evidenced by the decrease in weight borne by the injured leg (<50%) in WT mice. Similarly to mechanical allodynia, OA-induced weight-bearing deficit was significantly attenuated in both Par_2_^−/−^ and Par_2_Nav1.8 mice (Figure 7C). Par_2_^−/−^ global knockout mice had a significant prevention of weight-bearing deficit until day 14 while Par_2_Nav1.8 mice demonstrated a prevention until day 21 post OA induction. These data establish PAR_2_ as a key regulator of OA and demonstrate that its activation in sensory neurons (Par_2_Nav1.8 Model) is essential for OA-induced nociception.

**Figure 8:**
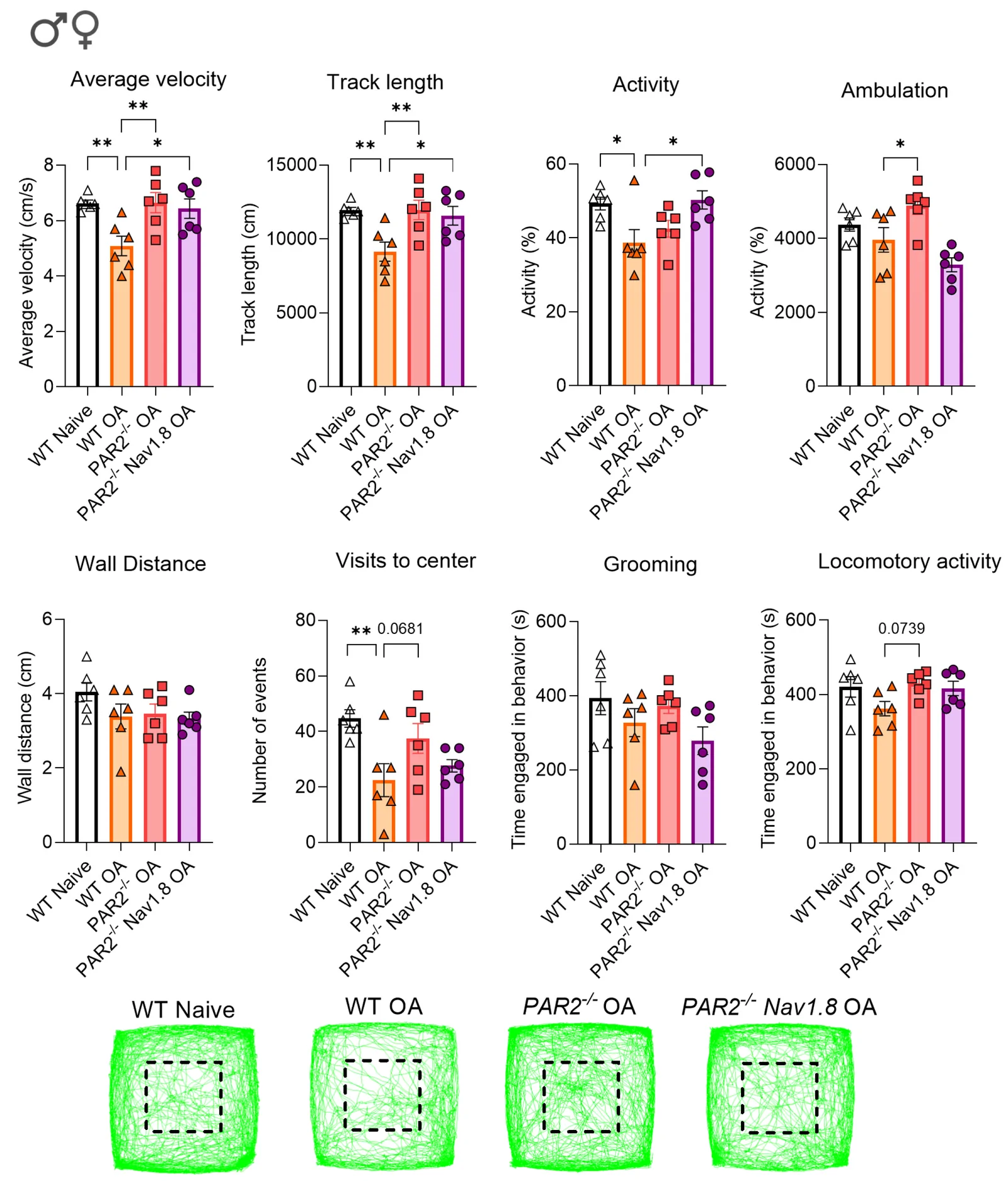
PAR_2_ participation in non-evoked behaviors induced by OA in mice. Non-evoked nociceptive behavior in OA wild type (WT), PAR_2_ global knockout (Par_2_^−/−^) and in mice lacking expression of PAR_2_ in Nav1.8 expressing neurons (Par_2_Nav1.8). Mice recorded for 30 min 8 days after OA induction. Visits to center area marked by a black square and representative images of the track records are shown. *n* = 6 mice/group. Data are expressed as the mean ± SEM. Data analyzed by 1-way ANOVA with Dunnett’s multiple-comparison test to determine differences between groups. * *p* ≤ 0.05, ** *p* ≤ 0.01 when compared with WT OA group.

To assess non-evoked pain behavior, mice were monitored in a behavioral spectrometer eight days post-OA induction. In WT mice, OA-induced nociception significantly reduced average velocity, track length, overall activity, and center visits. These deficits were largely abolished in Par_2_^−/−^ global knockout and Par_2_Nav1.8 mice. Notably, both knockout strains maintained average velocity and track length at levels comparable to baseline (Figure 8). These data show that the lack of PAR_2_ expression preserved locomotor parameters and exploratory behavior effectively preventing OA-induced impairments.

Overall, our findings indicate that PAR_2_ plays a significant role in OA-induced nociceptive behavior. Specifically, PAR_2_ expressed on synoviocytes and neurons contributes to OA inflammation, whereas the genetic deletion of PAR_2_ from sensory neurons reduces nociception. Importantly, we demonstrate that PAMAM-Chol-AZ3451-loaded nanoparticles are more effective than the free AZ3451 at inhibiting PAR_2_ signaling, highlighting the clinical potential of this targeted drug-delivery system.

## 4. Discussion

In the present study, we demonstrate that nanoparticle-encapsulated PAR_2_ antagonism represents a substantially more effective therapeutic strategy for OA pain than free drug administration. Using the MIA model of OA, we show that a single intra-articular injection of PAMAM-Chol-AZ3451 produced a prolonged and robust antinociceptive effect lasting up to 48–72 h, whereas free AZ3451 provided only transient relief within the first hours post-injection (Figure 1). Critically, this enhanced efficacy was attributable to two mechanistically distinct but complementary actions. The first is the suppression of PAR_2_-driven inflammatory cytokine release from synoviocytes (Figure 5C), and the second is the blockade of PAR_2_ signaling within Nav1.8-expressing nociceptors that innervate the knee joint (Figure 6). Together, these findings establish PAMAM-Chol-AZ3451 as a dual-targeted intervention capable of simultaneously blocking PAR_2_ activation that leads to both the inflammatory milieu of the joint and the neuronal signaling pathways that transduce OA pain [25].

The superior efficacy of PAMAM-Chol-AZ3451 reflects simultaneous action on two cell types and through cellular uptake to the endosomal compartment. Synoviocytes are central orchestrators of the inflammatory microenvironment in OA, producing cytokines and proteases that degrade cartilage and sensitize periarticular sensory neurons [35]. Our data confirm that SW982 human synoviocytes express functional PAR_2_, as evidenced by robust calcium mobilization following stimulation with the PAR_2_ agonist 2F-LIGRLO, and that this response was abolished by AZ3451 pre-treatment (Figure 5A,B). Consistently, PAMAM-Chol-Cy5 nanoparticles co-localized with EEA1-positive early endosomes within 2 h of incubation in these cells. PAR_2_ activation in synoviocytes resulted in increased release of a number of pro-inflammatory cytokines and chemokines which act as potent pro-inflammatory mediators that drive joint degradation [17,36,37]. Free AZ3451 and PAMAM-Chol-AZ3451 both significantly reduce IL-6 release relative to vehicle, indicating that the blockade of PAR_2_ is key in the release of IL-6 (Figure 5C,D). PAMAM-Chol-AZ3451 showed a distinct advantage for MIF, significantly reducing its release compared to both vehicle and free AZ3451. IL-6 is a potent mediator of arthritic pain while MIF has been associated with macrophage recruitment and acts as a bridge from the initial inflammatory response to the later destruction of the joint [36,38]. Notably, PAMAM-Chol-AZ3451 also significantly reduced IL-4 release compared to vehicle. This was unexpected given IL-4 is traditionally viewed as anti-inflammatory [39]. This may reflect a broader reduction in synoviocytes’ secretory activity following the endosomal inhibition of PAR_2_ signaling rather than a selective suppression of pro-inflammatory mediators and requires further investigation. Conversely, CXCL1/GROα release was significantly lower with free AZ3451 than with PAMAM-Chol-AZ3451, indicating that endosomal targeting does not uniformly enhance suppression across all PAR_2_-driven chemokines. CCL2/MCP-1 and C5/C5a showed reductions that trended in the expected direction but did not reach statistical significance. Increased C5/C5a expression has been linked to increased inflammation and tissue damage and may play a role in the shift from acute to chronic inflammation [40,41]. These findings suggest that inhibiting PAR_2_ signaling, and particularly targeting endosomal PAR_2_, disrupts the feed-forward loop linking joint inflammation to joint degradation and pain.

The ability of PAMAM-Chol-AZ3451 to inhibit the expression and release of these inflammatory factors suggests that PAR_2_ blockade in synoviocytes disrupts a key node in the feed-forward loop linking joint inflammation to peripheral sensitization.

Previous studies have demonstrated that endosomal PAR_2_ signaling is the driving factor in nociception and inflammation, and that targeting endosomal PAR_2_ signaling via nanoparticles is a more effective treatment [21,22,25]. Our results reinforce those findings and strongly support the concept that PAR_2_ continues to signal from endosomal compartments following receptor internalization, and that this intracellular signaling pool sustains cytokine production even after extracellular agonist is cleared. Accordingly, nanoparticles that are already present or co-traffic with PAR_2_ into endosomes are uniquely positioned to block this otherwise inaccessible cluster of activated receptors [21,23,24]. Consistent with this, intra-articular PAMAM-Chol-Cy5 was efficiently internalized by joint-innervating neurons (~59%, Figure 6B) in PAR_2_-muGFP reporter mice. In the OA-induced nociception model, PAMAM-Chol-AZ3451 was able to target endosomal trafficked PAR_2_ leading to the prolonged antinociceptive effect compared to free AZ3451 (Figure 1) [21,23], thereby confirming that nanoparticles can target activated and endocytosed PAR_2_ to attenuate signaling in PAR_2_-expressing sensory neurons. Together, the capacity of PAMAM-Chol-AZ3451 to attenuate synoviocyte cytokine release and to directly block PAR_2_ endosomal signaling on sensory neurons points to a dual-target mechanism that may explain its superior efficacy over free AZ3451 in reducing both the inflammatory burden and providing prolonged analgesic effect in pain-like behavior in vivo.

Genetic confirmation of PAR_2_ signaling in OA-induced nociception was provided by comparing Par_2_^−/−^ and Par_2_Nav1.8 knockout mice. Both lines showed significant reduction in mechanical allodynia and weight-bearing deficits throughout the OA time course. Additionally, OA-induced reductions in velocity, track length, and exploratory behavior in the behavioral spectrometer were largely abolished in both knockouts. Notably, Par_2_Nav1.8 mice showed protection extending to day 21, somewhat longer than the global Par_2_^−/−^ line, potentially reflecting compensatory inflammatory adaptations in non-neuronal tissues in the whole-body knockout (Figure 7). Taken together, these data establish that PAR_2_ on Nav1.8+ nociceptors is a key driver of OA pain and validate the targeting strategy of PAMAM-Chol-AZ3451 [9].

Sex is increasingly recognized as a critical biological variable in pain research, driving a paradigm shift toward testing both sexes in preclinical models and recent studies have increasingly identified distinct biological mechanisms driving nociception in males versus females [42,43]. In the MIA osteoarthritis model, evoked nociception in weight-bearing and von Frey tests was expressed equally between male and female mice. Interestingly, PAMAM-Chol-AZ3451 produced a more prolonged antinociceptive effect in females than in males. In contrast to evoked nociception, non-evoked behaviors assessed via the open field test revealed striking sexual dimorphism. While male OA mice exhibited expected pain-depressed activity, with reduced locomotion and anxiety-like behavior, female OA mice showed no significant changes in open field test. This divergence does not imply a lack of pain in females, as primary OA endpoints confirmed robust mechanical nociception and joint weight deficits. Rather, it highlights baseline behavioral differences, as female rodents naturally display higher baseline exploratory drive and locomotor activity, which can mask nociceptive alterations in open field tasks [44]. Because preclinical rodent studies relied almost exclusively on male subjects for decades, the affective and spontaneous behavioral manifestations of pain in females remain less defined. Overall, these findings underscore that while male and female mice experience equivalent joint-evoked nociception in the MIA model, they express distinct non-evoked behavioral phenotypes and pharmacological response profiles.

In conclusion, we demonstrate that nanoparticle-mediated intra-articular delivery of the PAR_2_ antagonist AZ3451 via PAMAM-Chol dendrimers provides superior and prolonged antinociceptive efficacy compared to free AZ3451 in a mouse OA model. This advantage stems from the ability of PAMAM nanoparticles to extend drug residence in the intra-articular space and deliver AZ3451 to endosomal compartments. By blocking intracellular PAR_2_ signaling in synoviocytes and sensory neurons, this approach suppresses cytokine release and inhibits the activation of sensory neurons underlying OA pain [16,19]. These findings establish PAMAM-Chol-AZ3451 as a proof-of-concept for endosomally targeted analgesic nanoparticles and provide a rationale for further development of this platform toward clinical translation in chronic joint pain.

## Data Availability

The raw data supporting the conclusions of this article will be made available by the authors on request.
